# Susac syndrome – different treatment approaches for one disease (analysis of case series)

**DOI:** 10.1007/s10072-025-08451-w

**Published:** 2025-10-27

**Authors:** Bogna Grygiel-Górniak, Maria Magdalena Joks, Łukasz Mazurkiewicz, Włodzimierz Samborski

**Affiliations:** 1 Department of Rheumatology, Rehabilitation and Internal Diseases, Poznan University of Medical Sciences, Poznan, 61-701 Poland; 2 Rheumatology Research Group, the Student Scientific Society of Poznan, University of Medical Sciences, Poznan, 60-701 Poland

**Keywords:** Susac syndrome, Branch retinal artery occlusion, Lesions in the corpus callosum, Sensorineural hearing loss

## Abstract

**Background:**

Susac syndrome is a rare, autoimmune, occlusive endotheliopathy that affects blood vessels in the central nervous system (CNS), retina, and inner ear. At initial medical evaluation, the classic triad of symptoms is present in less than one-third of patients, making diagnosis challenging.

**Methods:**

A brief review and retrospective analysis of the course, disease activity, exacerbations, and treatment of four patients with Susac syndrome.

**Results:**

The mean age of the patients was 25.8 years, and the average disease duration was 183.5 months +/- 90.65. The clinical triad was observed at the onset of the disease in only one patient. The first patient with predominant CNS symptoms was treated with prednisone, methotrexate, and sulfasalazine (IVIG and GCS i.v. were used during exacerbations). The next patient with exacerbation of ENT symptoms was treated with oral methylprednisolone (MP) and mycophenolate mofetil. The third patient had no clear predominance of any symptoms and responded well to azathioprine. The last patient presented mainly ophthalmological symptoms and was initially treated with cyclophosphamide (total dose 6200 mg) and IVIG. Later, azathioprine and mycophenolate mofetil were introduced.

**Conclusions:**

The described cases highlight differences in the course of the disease and response to immunosuppressive therapy. Patients initially responded very well to high doses of GCS and IVIG. However, in addition to long-term remissions, relapses also developed. Therefore, individual treatment based on the principles of personalized medicine is necessary.

## Introduction

Susac syndrome (SuS) is an autoimmune endotheliopathy affecting microvessels in the central nervous system (CNS), retina, and cochlea and thus can present with a triad of symptoms characterized by vision loss, hearing loss, and various neurological symptoms (including encephalopathy) [1–3]. Although the disease can develop in each age group, it primarily affects young women [4].

The diagnosis is based on cerebral, visual, and auditory symptoms. Ear, nose, and throat examinations often reveal newly diagnosed tinnitus, hearing loss, and peripheral vertigo consistent with vestibulocochlear involvement. Neurological abnormalities include headaches and changes in the corpus callosum on MRI [5]. Ocular changes are often characterized by partial or complete vision loss. Branch Retinal Artery Occlusion (BRAO), visible in Fundus Fluorescein Angiography (FFA), is noticed in 99% of patients [4]. Definite SuS is diagnosed when symptoms from all three organs are present; probable SuS covers symptoms from 2 organs, whereas possible SuS is suggested when one organ is affected. However, the clinical triad is very characteristic of SuS; it is estimated to occur in only 13% of patients at the disease’s onset [6]. Moreover, organ damage in the triad is often irreversible (especially concerning the inner ear) or curable in a narrow therapeutic window [7, 8]. The administration of proper treatment is crucial to prevent permanent disability. The pharmacological approach should include the disease’s severity and the organs affected [9].

Our case series describes four patients with different clinical manifestations of SuS. We collected information about the clinical course of the disease and compared the implemented treatment to current recommendations.

### Epidemiology

The incidence of SuS is underestimated and varies between 0.024 and 0.13 per 100,000. The syndrome is more common in women aged 20–40 (other data indicate 21–35), with a female-to-male ratio of about 3:1 [4, 10, 11] In the literature, the syndrome has also been described in pregnant women, and in such cases, pregnancy is considered high-risk [12].

SuS is often underdiagnosed or misdiagnosed because the typical triad is not observed at the onset of this entity. This is also the reason for the lack of accurate data on the incidence of the disease, which is due to nonspecific symptoms of varying duration and severity.

### Etiology

Currently, SuS is classified as an autoimmune endotheliopathy, microvessel destruction, and consecutive embolisms and microstrokes in the organs included in the triad of symptoms – the brain, inner ear, and retina [4]. Since the symptoms are not specific at the beginning of the disease, the differential diagnosis includes neuroinflammatory conditions, in particular, multiple sclerosis, stroke, migraine, and cerebral vasculitis [11].

### Clinical features

The classic triad of neurological, ophthalmological, and laryngological symptoms enables the diagnosis of SuS. Unfortunately, their absence at the onset of the disease delays diagnosis and appropriate treatment.

Depending on cerebral area involvement, patients with CNS symptoms suffer from various disorders [3]. Typical symptoms include migraine-like headaches, cognitive impairment, confusion, mental disorders including psychosis, memory loss, dementia, ataxia, hemiparesis, and cranial nerve disorders [3, 4, 13, 14].

Ophthalmological features usually include retinal artery occlusion (BRAO) or the development of small, punctate yellow-white plaques on the arterial wall called Gass plaques. Ophthalmological symptoms are characterized by vision loss or visual field reduction due to retinal changes [5, 14]. In addition, scintillating scotomas or photopsiae can be detected [15]. However, in many patients, the course of the disease may be asymptomatic, mainly at the beginning of the disease. Sensorineural hearing loss may be unilateral or bilateral, accompanied by tinnitus and vertigo, as capillaries in both the cochlea and the semicircular canals are affected [3, 14, 15]. The hearing loss is primarily noticeable in low to mid-tone range frequencies. However, impairment in higher frequencies is also possible [6, 7, 16]. The damages are usually irreversible, so permanent support for cochlear implants is needed.

## Diagnosis

Kleffner et al. proposed a diagnosis based on clinical findings in the central nervous system, eye, and ear [5]. The diagnosis of definite SuS is made when symptoms are observed in all three organs. If two criteria are met, the diagnosis of SuS is probable (one criterion suggests possible SuS). The diagnosis of definite SuS remains a challenge because the typical triad at the onset of the disease is relatively rare. A review by Dorr et al. describing 304 cases of SuS showed that only 13% of patients presented with clinical findings in all three organs at the onset of the disease [6]. The diagnosis of de novo SuS required confirmation of the current symptoms with appropriate imaging findings.

Neurological symptoms should be visualized using MRI, the standard examination for evaluating CNS lesions. Small, ischemic, hyperintense lesions visible on FLAIR and T2-weighted sequences occur in more than 80% of patients. Snowball-like lesions in the corpus callosum reflect multifocal microinfarcts and are typical for SuS (diagnostic criteria) [6, 7, 13]. In the subacute and chronic course of the disease, these lesions may transform into hypointense lesions in the corpus callosum visible on FLAR and T1-weighted sequences In addition, small multifocal lesions can be detected in the white matter –subcortically and periventricular. Lesions in the internal capsule create the “string of pearls” sign [4, 13]. Notably, no correlation is observed between the number and size of lesions and the severity of the disease [13]. MRI can be supported by cerebrospinal fluid (CSF) analysis, which may reveal elevated protein levels. The absence of oligoclonal bands helps differentiate SuS from multiple sclerosis [4, 13].

Ocular changes should be assessed by an ophthalmologist. Visual field examination, slit-lamp fundus examination, and fundus fluorescein angiography (FFA) are the methods of choice for diagnosing SuS [7]. Such diagnostics allow the detection of ischemic changes in the retina typical of branch retinal artery occlusion (BRAO). BRAO is one of the diagnostic criteria and occurs in 99% of patients [4, 6, 17]. Some patients can also observe clinically silent changes in the peripheral arteries. FFA allows the visualization of an atypical leakage pattern in the arterial wall, called arterial wall hyperfluorescence (AWH), located in or away from the occluded arteries [8, 17, 18]. Fundus imaging reveals small, yellow grass spots (GP) in the arterial bifurcations, which are highly specific for SuS but are not pathognomonic [3]. Coherence tomography angiography (OCTA), in turn, allows for the visualization of the thinning of the inner retinal layers while maintaining the normal condition of the outer retinal layers [4].

**Fig. 1:**
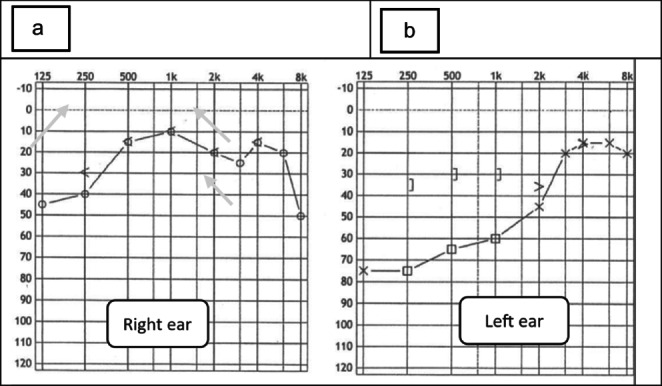
Audiogram: sensorineural lowering of the hearing level with predominant involvement of the middle and low tones

Each patient with hearing loss should undergo a pure tone audiometric test, which is necessary to confirm the presence of hearing impairment and assess its range (Fig. 1). Usually, such an analysis shows sensorineural hearing loss with a predominance of low and medium frequencies. Hearing loss can be unilateral or bilateral and is often irreversible [4, 6, 7]. Videonystagmography (VNG), including a vestibular organ color test, is performed if the patient complains of vertigo [5, 7].

## Case series

### Characteristics of the group

In Susacsyndrome, the complete triad of symptoms is not always present at the onset ofthe disease but develops over time. Similarly, in analysed cases, symptomsdeveloped over time and initially required differential diagnosis with otherneurological, ophthalmological, and otolaryngological conditions (Table 1).

**Table 1:** Characteristics of the group (anthropometric parameters and information about the course of Susac syndrome)

Analysed parameters	Patients with Susac syndrome			
	Patient 1	Patient 2	Patient 3	Patient 4
Age	45	37	38	43
BMI > 25 kg/m2	Yes	No	Yes	yes
Gender	Male	Female	Female	Male
Disease duration (months)	146	120	318	150
Patient’s age at the first symptom	33	27	12	31
Time from first symptoms to diagnosis of the disease (months)	1	3	5	5
Symptoms at the onset of the disease	• headache, vertigo • paresthesia • unstable balance • problems with short-term memory • disorientation in time • amnesia • MRI: macular and ovoid lesions in both cerebral hemispheres • tinnitus and sensorineural hearing loss (75 dB) • FFA: BRAO • multifocal arteriolar disease in the retinal periphery	• headache • cognitive impairment • hearing loss	• headache • gait disorder • imbalance	• numbness of the right part of the face, hands, and thorax • MRI: periventricular lesions in the corpus callosum • visual field loss in the right eye (FA - BRAO) • transient • hearing loss (~ 30dB)
Typical triad at the onset of the disease	Yes	No (developed 4 years later)	9 (developed 23 years later)	Yes
Number of flares	6 (within 14 years)	5 (within 8 years)	9 (within 25 years)	11 (within 12 years)

### Patient number 1

**Fig. 2:**
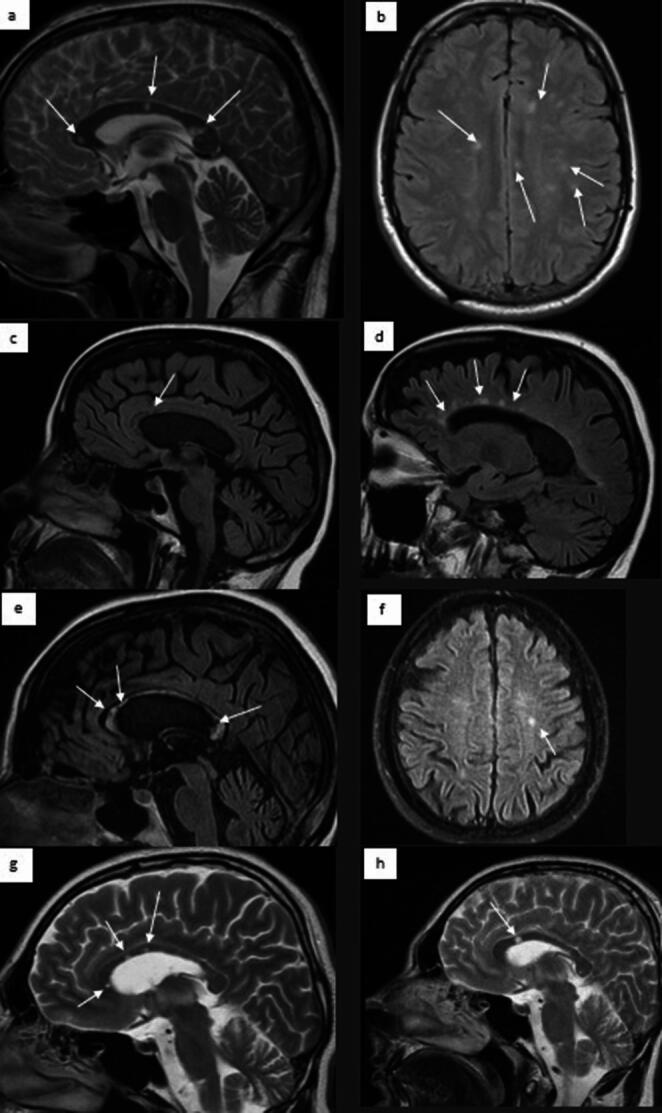
MRI scans of analyzed patients. Patient 1: Sagittal PD + T2: 2a. Multiple hyperintense foci in the atrophic corpus callosum; 2b. Axial PD + T2: Multiple confluent hyperintense lesions bilaterally in the white matter of the frontal and parietal lobes; **Patient 2**: Sagittal T2, FLAIR: 2c multiple lesions merging with blurred contours in narrow and hypotrophic corpus callosum, 2 d scattered, small, numerous hyperintense lesions up to 7 mm in the periventricular white matter and corpus callosum that are perpendicular to the ventricular body. **Patient 3**: Sagittal T2 FLAIR: 2e. Atrophic corpus callosum atrophic with numerous small hyperintense and malacial foci; Axial T2 FLAIR 2 f. Small (diameter up to 5 mm) hyperintense foci in the subcortical white matter of both semi-oval centers and the left temporal lobe. **Patient 4**: Sagittal T1-weighted MRI: Ovoid lesions in hypotrophic corpus callosum (2 g and 2 h)

The first case describes a 45-year-old man who complained in 2012 of neurological and laryngological symptoms. (vertigo, paresthesia in the tongue area, irritability, drowsiness, tinnitus, and hearing loss in the left ear – 75 dB of hearing loss in the audiometry).After two months, the neurological symptoms returned with additional balance disorders, problems with short-term memory, and cognitive impairment. The patient was referred to the Neurology Clinic with suspected meningitis. He was given glucocorticosteroids intravenously, and his condition improved. Unfortunately, the symptoms returned after a week, and he was readmitted to the Neurology Clinic. Electroencephalography (EEG) revealed theta waves in the anterior leads. In August 2016, MRI showed ovoid lesions in both cerebral hemispheres (Fig. 2a and b) and multiple sclerosis was suggested. Treatment with mannitol and methylprednisolone was initiated, which resulted in temporary improvement.

Three months later, he developed drowsiness, confusion, impaired concentration and memory, hearing loss in the left ear, and neck stiffness. A typical BRAO pattern was detected in the FFA. Audiometry showed mild sensorineural hearing loss The patient was diagnosed with Susac syndrome (as described before) [19]. Prednisone treatment (20 mg/day) was initiated, which resulted in an improvement in clinical symptoms. In 2015, due to recurrent inflammation of the small joints of the hands, the patient was treated with chloroquine; however, no improvement was observed, and thus, methotrexate (MTX) was implemented with good clinical effect.

Laboratory tests showed hyperglycemia, hypercholesterolemia, and ANA antibodies (titer 1/80). Contracture of the right elbow joint and proximal interphalangeal (PIP) joints of the third finger of the right hand were observed. In 2018, the patient reported recurrent pain and swelling of the index fingers. NSAIDs were added to methotrexate, and physiotherapy was recommended. Despite the intensification of treatment, no clinical improvement was observed. Therefore, diagnostics were expanded, which allowed the detection of Yersinia infection, and the treatment was modified (the patient received hydroxychloroquine, sulfasalazine, and prednisone). Currently, his general condition is stable, and he is on prednisone treatment at a dose of 2.5 mg/day with a further plan of steroid tapering. No further progression of changes is observed in the MRI.

### Patient number 2

The second case concerns a 37-year-old woman who complained of headaches, cognitive impairment, and sudden hearing loss in April 2014 (Table 2). The patient was hospitalized in a rheumatology clinic, where she was diagnosed with Susac syndrome. She was initially treated with prednisone 60 mg and mycophenolate mofetil (MMf) 500 mg/day, which had a good clinical effect.

**Table 2:** The course of the diseases and treatment in patients with Susac syndrome

	The analysis of clinical, biochemical, serological data and imaging studies of patients with Susac syndrome				
	Time	Patient 1	Patient 2	Patient 3	Patient 4
Period of observation	months	118	120	85	150
Neurological symptoms	at the onset	• headache, vertigo, paresthesia • unstable balance • problems with short-term memory • disorientation in time	• headache • cognitive impairment • hearing loss	• headache • gait disturbance • imbalance	• numbness in the right side of the face, hands, thorax
	flare	• consciousness impairment • paresis • drowsiness • disorientation • concentration • memory disorders	• headache • cognitive impairment	• headache • vertigo • tremor of the head and trunk	NA
	currently	NA (patient is under control in outpatient clinic)	• occasionally vertigo • mild headache	headache, vertigo tremor of the head and trunk	NA (patient is under control in outpatient clinic)
Ocular symptoms	at the onset	BRAO multifocal arteriolar disease in the retinal periphery	-	amaurosis fugax	visual field loss in the right eye
	flare	NA	active neuropathy of ocular nerve	changes typical for BRAO	visual field loss in the right eye later, scotoma in the peripheral vision of the left eye
	currently	NA	chronic neuropathy of ocular nerve	NA	NA
Otolaryngological symptoms	at the onset	hearing loss	sudden hearing loss of the right ear	hearing loss	transient hearing loss
	flare	hearing loss of the left ear	deafness of the right ear (2016); lesions in the membranous labyrinth; partial hearing loss of the left ear	hearing loss	hearing loss
	currently	-	Chronic bilateral hearing loss	chronic hearing loss	chronic hearing loss
Other symptoms	at the onset	pain of MTP and knee	-	Th and LS spine pain caused by OA	NA
	flare	joints pain	-	Th and LS spine pain	NA
	currently	NA	-	Th and LS spine pain	NA
ESR [mm]	at the onset	2	6	10	3
	flare	4	6	38	8
	currently	4,7	10	4	4
CRP [mg/l]	at the onset	2,77	0,1	2,5	2,17
	flare	15,1	6,1	58,2	135,6
	currently	4,7	3,7	2,6	5,2
WBC [x103/mm3]	at the onset	7,7	10	11,6	5,21
	flare	7,8	8,7	7,3	11,9
	currently	8,7	8	7,3	4,85
Hb [g/dl]	at the onset	15,2	14,4	14,4	14,6
	flare	14,3	14,1	11,8	10,4
	currently	15,1	10,8	13,1	14,8
PLT [x103/mm3]	at the onset	273	293	296	254
	flare	276	293	324	259
	currently	317	411	289	230
Vitamin D [ng/ml]	at the onset	NA	NA	18,8	Nd
	flare	NA	NA	30,23	30,8
	currently	33,7	81,4	35,8	38,72
Glucose profile [mg/dl]	at the onset	92	82,5	86,3	91,1
	flare	93	82,5	83,9	93,4
	currently	101,8	83,6	80,3	86,4
Serological analysis ANA	at the onset	not detected	ANA 1/80 (speckled and homogenous); ANA profile: PCNA, dsDNA; Pm-Scl 100	not detected	not detected
	flare	homogenous and speckled, 1/160	ANA 1/80 (speckled and homogenous); ANA profile PM100; Pm-Scl 100, PCNA	homogenous, 1/80	not detected
	currently	speckled type, 1/80, Pm-Scl100, PCNA	ANA 1/80 (speckled); ANA profile: Pm-Scl 100, PCNA	Speckled, 1/80, PM-Scl100	speckled, PM – Scl100
MRI	at the onset	macular and ovoid lesions in both cerebral hemispheres	NA	NA	periventricular lesions in the corpus callosum
	flare	progression of disease • multiple confluent hyperintense lesions located bilaterally in the white matter of the frontal and parietal lobes, • multiple hyperintense foci in the atrophic corpus callosum.	• scattered, small, multiple hyperintense lesions up to 7 mm in the periventricular white matter and corpus callosum that are perpendicular to the ventricular body • lesions in the matter and the membranous labyrinth • s narrow and hypotrophic corpus callosum is	thinning of the corpus callosum • atrophic corpus callosum with numerous small hyperintense and malacial foci • small hyperintense foci in the subcortical white matter of both semi-oval centers and the left temporal lobe	• Narrow corpus callosum with numerous pathological hyperintense foci. • bilateral small hyperintense foci in the radial coronae of both frontal lobes.
	currently	no further lesions were detected	• small lesions in the white matter, which merge with each other and have blurred contours • no changes in the membranous labyrinth (stable state and comparable to previous analysis)	compared to previous imaging	hypotrophy and periventricular lesions in the corpus callosum
FFA	at the onset	BRAO	-	BRAO	BRAO
	flare	BRAO	neuropathy of II nerve and retina, possibly due to BRAO	BRAO	BRAO, CRAO, blockage of the distal arch of the inferior temporal retinal artery
	currently	BRAO	neuropathy of II nerve and retina; BRAO	BRAO	BRAO, CRAO, blockage of the distal arch of the inferior temporal retinal artery
Audiometry	at the onset	sensorineural hearing loss of left ear (75 dB)	sensorineural hearing loss of the right ear	sensorineural hearing loss	transient sensorineural hearing loss (30 dB)
	flare	sensorineural hearing loss (40 dB)	bilateral sensorineural hearing loss (more extensive in the right ear)	sensorineural hearing loss	sensorineural hearing loss
	currently	chronic sensorineural hearing loss	chronic bilateral sensorineural hearing loss, which requires hearing aids	chronic sensorineural hearing loss	chronic sensorineural hearing loss
GCS	at the onset	prednisone 20 mg/day	prednisone 60 mg/day	dexamethasone 8 mg p.o. MP 3 × 500 mg i.v., later 12 mg p.o.	initially MP 3 × 500 mg i.v., later 32 mg p.o. with decreeing dose (slowly withdrawn after osteoporosis development)
	flare	MP 3 × 500 mg i.v., later prednisone 10 mg/day p.o.	MP 3 × 500 mg i.v., later prednisone 25 mg/day p.o.	MP 3 × 500 mg i.v., later 20 mg/day p.o.	MP 3 × 500 mg i.v., later 32 mg slowly reduced to 8/10 mg/day p.o.
	currently	prednisone 2,5 p.o. mg/day	MP 6 mg/day p.o.	MP 3 × 250 mg i.v.; later 6 mg/day p.o.	MP 4 mg/day p.o.
Immuno-suppressants	at the onset	-	MMf – 500 mg/day	AZA − 100 mg/day	AZA 100 mg/day
	flare	HCl 250 mg/day 2015–2016 (due to joints inflammation) MTX – 15 mg/week (2016–2018) HCI – 200 mg/day (2019–2020) SFN – 2000 mg/day (2020 till now)	MMf 500 mg/day (higher dose was not tolerated)	AZA 100 mg/day	CYC – total dose 6200 mg AZA 100 mg/day MMf − 3 g/day
	currently	-	MMf 500 mg/day (intolerance of higher doses)	AZA 100 mg/day	MMf 3 g/day
IVIG	at the onset	Yes	Yes	Yes	Yes
	flare	Yes	Yes	Yes	Yes
	currently	-	-	-	Yes
iatrogenic effects (GCS-induced)	at the onset	-	-	Weakness of lower extremities (12 h)	mixed hyperlipidemia, aseptic bone necrosis
	flare	-	-	-	mixed hyperlipidemia, aseptic bone necrosis
	currently	Hyperglicemia, hypercholesterolemia	hypercholesterolemia, higher glucose level	hypercholesterolemia	mixed hyperlipidemia, aseptic bone necrosis
Osteoporosis	at the onset	-	-	-	Yes
	flare	-	-	-	Yes
	currently	-	-	-	Yes

*AZA* azathioprine, *MTX* methotrexate, *MP* methylprednisolone, *SFN* sulfasalazine, *CYC* cyclophosphamide, *MMF * mycophenolate mofetil, *HcL *hydroxychloroquine, *BRAO *branch retinal artery occlusion, *CRAO *central retinal artery occlusion, *MTP *metacarpophalangeal, *FFA *fundus fluorescein angiography, *No *not present, *ImmS *immunosuppressants, *Th *thoracic, *LS *sacroiliac, *OA *osteoarthritis, *NA *not available

In August 2016, sudden recurrent bilateral hearing loss was observed. The patient was admitted to the Audiology Department, and an MRI of the brain showed a white matter lesion with concurrent inflammation of the internal carotid arteries and changes in the membranous labyrinths (Fig. 2c and d). During hospitalization, the patient received intravenous methylprednisolone, vitamin B, and hyperbaric oxygen therapy; however, no improvement was observed. Therefore, since October 2016, she has been treated with an increased dose of prednisone 25 mg/dayand mycophenolate mofetil 500 mg/day. The steroid dose was gradually reduced by 2.5 mg per week until the drug was discontinued; MMF was taken continuously. The patient’s condition remained stable until 2017, with persistent symptoms such as hearing loss, dizziness, and headache.

FFA performed in 2018 showed partial retinal and optic nerve atrophy with BRAO, and the patient was treated with intravenous methylprednisolone. After the mycophenolate mofetil, the patient reported nausea and reduced the dose of the medication herself to 500 mg/day (higher doses of mycophenolate mofetil and other immunosuppressants caused side effects). Recent biochemical studies showed mild normocytic anemia and hypercholesterolemia. The patient is currently receiving methylprednisolone 6 mg daily with a low dose of mycophenolate mofetil, and her condition is stable.

### Patient number 3

The third case regards a 38-year-old woman with worsening neurological symptoms. The first symptoms, such as headache, gait disturbances, and balance disorders, appeared in 1998 (Table 2). The tests at that time revealed demyelination in MRI in the corpus callosum and periventricular white matter of the brain (Fig. 2e and f). Antibiotics and glucocorticosteroids used resulted in temporary improvement of the observed symptoms. In 2008, the neurological symptoms returned, and acute disseminated encephalitis with tetraplegia was suspected. In 2012, in addition to the neurological symptoms, muscle weakness and ocular symptoms (amaurosis fugax) also developed, and the patient was diagnosed with multiple sclerosis (MS).

Five years later, the patient was hospitalized in the Rheumatology Clinic, and Susac syndrome was diagnosed. She complained of weakness of the lower limb muscles and ocular symptoms (increased lacrimation and recurrent eye fatigue during reading). FFA showed BRAO. Clinical improvement was observed after administering immunoglobulins and methylprednisolone intravenously for three days. The treatment was continued with oral glucocorticosteroids and azathioprine. In 2021, hearing loss in the left ear and ischemic retinal changes in the left eye were noted. The patient received methylprednisolone 500 mg intravenously for three consecutive days, which was later replaced with oral glucocorticosteroids.

Recently, the patient complained of headaches, dizziness, excessive eye lacrimation, and pain in the thoracic and lumbar spine. Physical examination revealed head and trunk tremors and limited right hip mobility. Hypercholesterolemia and ANA PM-Scl100 antibodies were detected. The patient received methylprednisolone pulses intravenously. Improvement in the clinical condition was observed. She continued treatment with methylprednisolone (6 mg/day) and oral azathioprine, and her condition has been stable.

### Patient number 4

The last case concerns a 43-year-old man who was admitted to the rheumatology clinic in April 2024 with a diagnosis of SuS and vision problems. The first symptoms appeared in 2012 with a sudden visual field loss in the right eye. Fundus examination showed cotton wool spots in the right eye, and FFA confirmed BRAO. In addition, two thrombotic changes were observed in the left retina and one in the right retina. The visual impairment was associated with transient hearing loss. The audiogram showed a 30 dB decrease in the sensorineural hearing level.

Two months after the visual impairment, the patient complained of numbness on the right side of the face, hand, and chest. MRI showed ovoid and periventricular lesions in the corpus callosum (Fig. 2g and h). The diagnosis of SuS was confirmed, and the patient was treated with intravenous glucocorticosteroids and intravenous immunoglobulin administration (IVIG). From 2012 to 2014, the treatment was conducted using oral glucocorticosteroids and azathioprine. The thrombophilia was diagnosed (increased factor VIII), and the patients were treated with clopidogrel (as described before) [20]. Since recurrent ocular symptoms predominated in 2014, the patient was treated with cyclophosphamide with clinical improvement.

Unfortunately, in 2016, the patient’s vision deteriorated further, and a fluorescent retinal artery examination revealed a blockage of the distal arch of the inferior temporal retinal artery. Laser therapy was performed, which resulted in improved vision. In 2019, the patient complained of scotomas in the peripheral field of vision of the left eye. Tests showed ischemia caused by a blockage of the central retinal artery (CRAO) in the left eye without symptoms of vasculitis. MRI revealed hypotrophy of the corpus callosum. The patient was hospitalized, and treatment with azathioprine and methylprednisolone (intravenously and orally) was initiated. Further hospitalizations took place in 2021 and 2023 - MRI of the brain did not show further changes, and azathioprine treatment was continued. Unfortunately, in the course of glucocorticosteroids therapy, osteoporosis and aseptic bone necrosis of the hips and arms developed, and the patient required endoprosthesis of both hips.

The patient was consulted in an ophthalmology clinic, where a new ischemic focus was found in the right eye. Methylprednisolone pulses were administered, azathioprine was discontinued, and mycophenolate mofetil was introduced. Currently, the patient complains of pain in the right hip (caused by the injury). Laboratory tests showed slightly increased CRP, LDH, ANA antibodies 1:80, speckled staining - PM-Scl100. The patient received methylprednisolone pulses, and ocular and musculoskeletal symptoms were improved. Chronic treatment with mycophenolate mofetil (3 g/day) and methylprednisolone (8 mg/day) with slow reduction of the dose was continued. Previous repeated attempts to discontinue glucocorticosteroids have shown a recurrence of symptoms at doses below 4 mg/day.

## Discussion

In the literature, various clinical symptoms are described at different stages of the disease. In addition, specific microangiopathy of one organ (e.g., brain, eye, and cochlea) often dominates. Therefore, the clinical course is very variable at the time of diagnosis. Data from the literature show that the typical triad for SuS occurs in 13–30% of all patients at the beginning of the disease, which delays diagnosis [6, 8, 21]. Similarly, in our study, the typical triad occurred in only two patients (patients no. 1 and no. 4). Moreover, the predominance of neurological, ocular, or laryngological symptoms causes the lack of visible patterns of organ involvement. Specific symptoms usually appear quickly and are reversible only in the minimal therapeutic window (unfortunately, they are more often irreversible) [4, 7, 8].

**Fig. 3:**
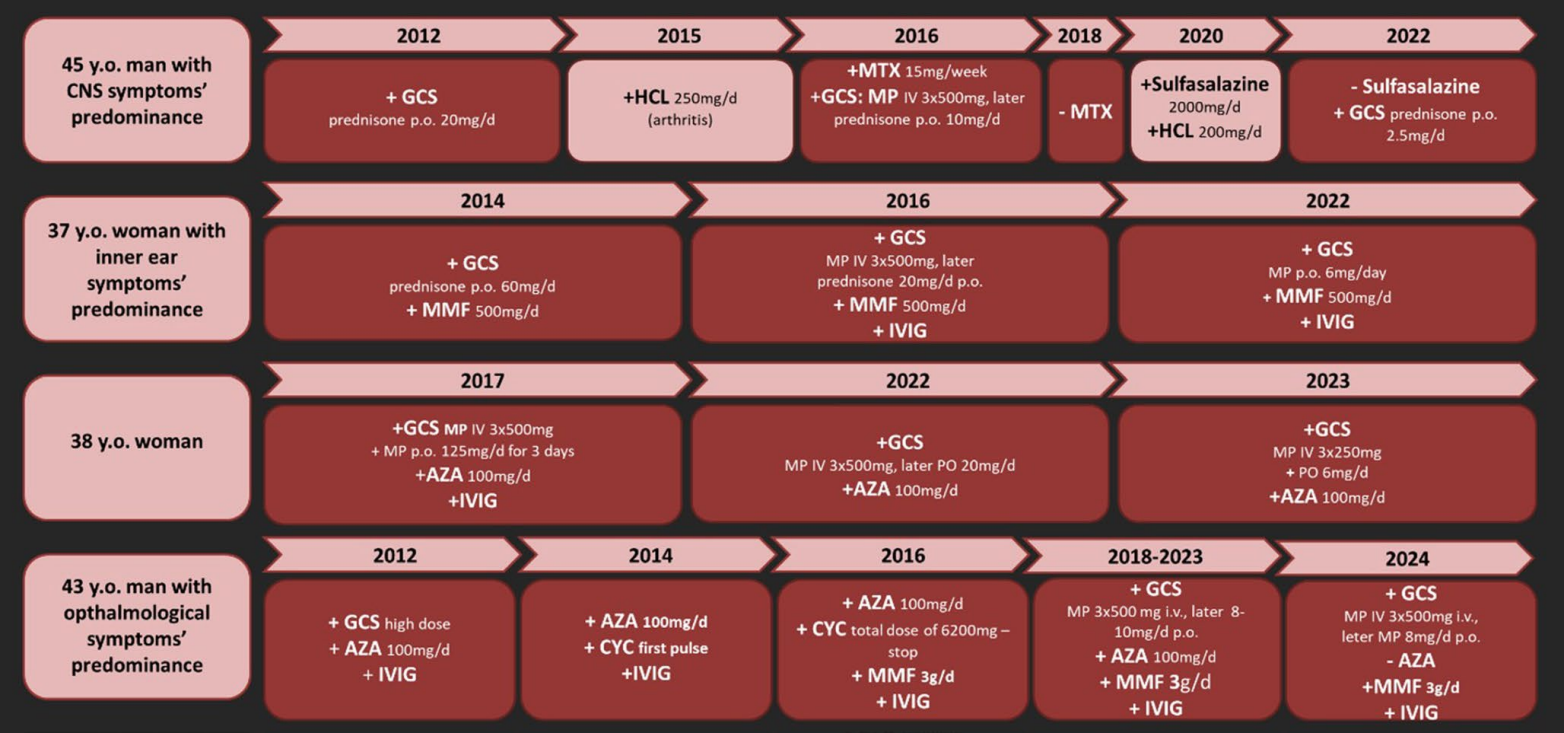
Graphic presentation of the treatment applied to our patients within 10-years observation from 2012 to 2022. Specific medications and modifications of the treatment are included in this figure. HCL – hydroxychloroquine; MTX – methotrexate; MMF – mycophenolate mofetil; IVIG – intravenous immunoglobulins; AZA – azathioprine; CPM – cyclophosphamide

The first symptoms are usually observed in young adults aged 16 to 40, with an average age of 31 [6, 22, 23]. However, the age of onset ranges from 7 to 72 years. Therefore, prompt implementation of appropriate therapy is of great importance. Considering the different clinical backgrounds, there is a need for patient-tailored treatment according to therapy standards (Fig. 3). Choosing the right pharmacological approach is as important as it is difficult.

Due to the low incidence of the disease and the lack of randomized studies, case series and retrospective studies remain the only source of information on treatment [14]. Since similarities have been observed between the immunopathogenesis and clinical course of SuS and juvenile dermatomyositis (JDM), the JDM treatment regimen is often used in patients with SuS (primarily pediatric patients) with good clinical outcomes [8, 9]. A similar treatment regimen has been described in the case of a 16-year-old girl diagnosed with SuS who was treated according to the JDM recommendations (methylprednisolone, cyclophosphamide pulses, and IVIG infusion), resolving the symptoms [24]. Among the many attempts to find suitable biomarkers for SuS relapse and remission, the study by Plantone et al. provides two biomarkers associated with neuronal and glial degeneration – neurofilament light chain (NfL) and glial fibrillary acidic protein (GFAP). Serum levels of these biomarkers are significantly increased in patients with severe SuS compared to the control group. Interestingly, SuS relapse is associated with a significant increase in NfL levels, while GFAP levels do not increase as dynamically. Therefore, NfL and GFAP are considered biomarkers used to monitor disease relapse and severity. However, further studies are needed to apply both proteins in everyday clinical practice [25].

Glucocorticosteroids, immunosuppressive drugs, IVIG infusions, and acetylsalicylic acid are the cornerstones of treatment. However, the choice of drugs and dosage depends on the severity of the disease and the predominance of neurological, ocular, or ENT symptoms. In 2018 Rennebohm et al. collected 110 patients diagnosed with definite/probable SuS and proposed treatment guidelines [9]. According to current knowledge, moderate and severe SuS with predominant CNS symptoms require intravenous methylprednisolone pulses (1000 mg daily for 3–7 days). Treatment should be continued with oral prednisone at a dose of 1 mg/kg/day, which is slowly reduced to 5 mg/day [8, 9].

In our report, after receiving the SuS diagnosis, each patient was immediately treated with GCS according to current treatment recommendations for this disease. Unfortunately, some of our patients were initially misdiagnosed with meningitis or multiple sclerosis, which delayed appropriate treatment.

Current literature suggests that concomitant IVIG (2 mg/kg for two days, every two weeks) prevents rapid and acute relapses during glucocorticosteroids cessation. Therefore, IVIG is recommended at all stages of the disease, especially in severe SuS or retinal damage [8, 9, 26]. IVIG treatment should be continued for at least 12 months. Kleffner et al. reported 2 cases of women treated with subcutaneous immunoglobulin who showed good tolerance to such therapy and improved their general condition [27]. Although subcutaneous administration is rarely used in clinical practice, it is worth considering as an alternative to IVIG; however, it should be emphasized that there is still insufficient data on such treatment. Furthermore, acetylsalicylic acid should be initiated at the beginning of the disease due to its antiplatelet and anti-inflammatory effects [14]. Thus, initial therapy with glucocorticosteroids, IVIG, and acetylsalicylic acid has been considered the mainstay of the therapeutic approach in SuS.

Cyclophosphamide or rituximab treatment is reserved for severe cases [26]. For example, cyclophosphamide is the first-line drug in cases of severe CNS involvement [8, 24]. Initially, two cycles of intravenous cyclophosphamide (10–15 mg/kg, max. 1200 mg) over two weeks are recommended. Later, such therapy can be continued with longer intervals between infusions. In our analysis, only patient no. 4 required cyclophosphamide infusion due to recurrent ocular symptoms. Since cyclophosphamide has many side effects, oral immunosuppressive therapy with another drug should be continued after its administration. Most recommendations suggest mycophenolate mofetil in monotherapy or with tacrolimus for chronic treatment. However, azathioprine is also a promising therapeutic option [9, 14]. Furthermore, combined cyclosporine therapy and mycophenolate mofetil as first-line treatment appears to have a good clinical effect [23, 28].

Recent data also emphasize the efficacy of methotrexate. This drug can be used as monotherapy or added to azathioprine or mycophenolate mofetil (especially after disease relapse) [14, 23]. Since methotrexate has a good clinical effect in many autoimmune diseases, this drug can be used in SuS. In our report, patient no. 1 was initially treated with methotrexate and obtained a good clinical response. However, this drug had to be discontinued due to childbearing plans. Since the disease manifests itself mainly in young people of reproductive age, the potential teratogenic effect should be considered [23, 28].

In SuS, the use of rituximab (anti-CD20 monoclonal antibody) has also been described in the literature. Current recommendations suggest early administration of rituximab only in severe disease with dominant CNS involvement or in patients with relapses. The initial dose of rituximab 1000 mg should be repeated after 14 days and then every six months [8, 9, 14, 29]. In addition to rituximab, single studies using anti-TNF-alpha inhibitors have been described. They have shown promising results, but further studies are needed to confirm the beneficial long-term outcome [30, 31].

### CNS microangiopathy

The initial symptoms of SuS may result from microangiopathy occurring in only one organ. This situation was observed in patient no. 1, who presented only CNS symptoms. Because the neurologist initially suspected infectious meningitis, intravenous methylprednisolone was not initiated. After ruling out the viral origin of the neurological symptoms, oral prednisone (20 mg/day) and IVIG were initiated with a good clinical response. Unfortunately, during the disease, the patient reported chronic neurological disorders and recurrent joint pain. Musculoskeletal symptoms responded very well to methotrexate treatment. However, methotrexate was discontinued due to reproductive plans. After the diagnosis of yersiniosis, sulfasalazine and prednisone were introduced. After several months of treatment, the general good condition allowed sulfasalazine to be discontinued. Since 2020, no disease flares have been observed.

According to the treatment guidelines of Rennebohm et al. [9] and Bose et al. [14], almost all neurological symptoms of SuS, regardless of their severity, should be treated with methylprednisolone pulses (1000 mg/day for 3–7 days) followed by oral prednisolone (1 mg/kg for 4 weeks). IVIG is recommended for the gradual reduction of glucocorticosteroids. In addition, immunosuppressive therapy (mycophenolate mofetil, cyclophosphamide, tacrolimus, and rituximab) is used to prevent disease exacerbations. Our patient no. 1, with predominant CNS symptoms, received methylprednisolone pulses during exacerbations at a lower dose than recommended, with a good clinical outcome. Unfortunately, initial intravenous steroid therapy was delayed due to a misdiagnosis of viral encephalitis. After the correct diagnosis was made, the choice of immunosuppressive therapy was discussed with the patient as a joint decision between the physician and the patient. In this case, the type of therapy depended on the patient’s reproductive plans (withdrawal of methotrexate) and comorbidities (hydroxychloroquine initiated due to arthritis, sulfasalazine after the diagnosis of yersiniosis). Our patient did not receive rituximab because he responded well to conventional immunosuppressive drugs. Furthermore, reports of the efficacy of RTX in SuS appeared after the final diagnosis was made and after the initiation of effective treatment.

### Ocular microangiopathy

The most typical ocular microangiopathy is BRAO, which responds well to immunosuppressive drugs, including glucocorticosteroids. Steroids are usually used systemically (orally or intravenously), and the dose depends on the severity of the disease. If the disease is stable (no new changes in FFA control), the treatment can be gradually tapered over the next six months [17]. Glucocorticosteroids can also be administered locally. For example, Yepez et al. reported a case of intravitreal triamcinolone in patients with SuS, which led to clinical improvement [32]. Severe CNS symptoms are treated with methylprednisolone pulses followed by oral prednisone, which is the initial treatment and remains a sufficient pharmacological approach in the absence of neurological symptoms [10, 17]. Recommendations for the treatment of ocular symptoms are similar to those for the treatment of CNS symptoms (methylprednisolone in pulse doses of 1000 mg/day for 3 days). However, the dose of oral prednisolone is lower, ranging from 40 mg to 60 mg/day for 1–2 weeks. The steroid dose is then gradually reduced, and rituximab is added to patients with a poor response to treatment. In very severe cases with an increased risk of vision loss, polytherapy is used, including cyclophosphamide or tacrolimus [9].

A severe ophthalmological manifestation of SuS was observed in patient 4. The typical triad was present at the beginning of the disease, but ophthalmological symptoms dominated during periods of exacerbations (reduced visual field, scotomas, decreased acuity)—initial treatment with i.v. methylprednisolone (3 × 500 mg pulses in the beginning and during flare-ups), IVIG, and cyclophosphamide pulses (total dose of 6200 mg) resulted in clinical improvement, and ocular symptoms decreased (at least partially). Glucocorticosteroids was continued with oral prednisone in combination with other immunosuppressive drugs.

Unfortunately, this patient developed multiple complications due to steroid therapy, such as osteoporosis, hyperlipidemia, and aseptic osteonecrosis, which required surgical intervention (hip and shoulder endoprosthesis). Azathioprine was introduced after cyclophosphamide treatment, which resulted in only transient stabilization, and the patient required mycophenolate mofetil treatment. Recent data show that adding mycophenolate mofetil to glucocorticosteroids therapy prevents relapses during prednisone reduction. Therefore, this medication should be considered at the onset of the disease and is recommended in the Rennebohm’s guidelines [8, 9, 26].

This patient received IVIG from the first to the current hospitalization with good clinical effect. IVIG contains antibodies from many healthy donors, which can stabilize immunological processes and reduce inflammation. After binding of host complement to the Fc fragment of IVIG, inhibition of the complement cascade is observed [33]. Therefore, IVIG is used in SLE, idiopathic inflammatory myopathies, ANCA-associated vasculitis (AAV), SuS, and various inflammatory demyelinating polyneuropathy occurring in rheumatic disease [33, 34].

The treatment regimen used in patient 4 was similar to the recommended therapy for retinal vasculopathy in SuS [9]. However, in his case, the adverse effects of aggressive steroid therapy were observed, and consequently, the steroid dose had to be adjusted to the amount tolerated by the patient. Unfortunately, despite initial improvement in the patient’s condition, exacerbations were observed when steroids were reduced below 5 mg/day prednisone equivalent. Azathioprine was not sufficient to prevent exacerbations. Therefore, it was changed to mycophenolate mofetil.

According to ophthalmological standards, BRAO should be monitored with repeated control FFA. Such monitoring was performed in all patients discussed. In neovascularization, anti-VEGF therapy (anti-vascular endothelial growth factor) or laser photocoagulation should be used to prevent intraocular bleeding [8, 17]. Fortunately, none of our patients present such a complication.

Patient 3, who presented with mainly ophthalmologic and CNS symptoms, provides excellent evidence that SuS symptoms are similar to those of demyelinating diseases. She was initially misdiagnosed with acute disseminated encephalomyelitis (ADEM) because of headaches and gait disturbances. She was subsequently diagnosed with MS after an episode of amaurosis fugax and muscle weakness. MRI scans performed at the time of diagnosis revealed demyelinating changes in the corpus callosum and periventricular white matter. These changes can occur in both MS and SuS. The differences between MRI scans and the symptoms of SuS and other vasculitides and demyelinating diseases are sometimes subtle (Table 3). Brain biopsy is rarely performed due to its invasiveness, difficult access, and risk of complications. The clinical features and FFA findings in patient 3, which evolved over time, led to the diagnosis of SuS. Unfortunately, the lack of a complete triad of symptoms at the onset of the disease often makes the diagnosis difficult.

**Table 3:** Differential diagnosis of Susac syndrome

	Differential diagnosis of SuS				
	Disease	MRI – lesions’ localization in CNS	Characterization of lesions in the CNS	Symptoms	Reference
Vasculopathy	Susac Syndrome	Corpus callosum, internal capsule, Grey matter, leptomeningeal arteries	• corpus callosum: thinning, “snowballs”, lesions evolving into “wholes” • internal capsule: “string of pearls” sign	• headache • memory loss • loss of concentration • gait disturbances	[8]
	Primary Central Nervous System Vasculitis (PCNSV)	White and grey matter	• infarcts • hyperintensities • hemorrhagic lesions	• headache • strokes • cognitive impairment • personality changes	[35]
	Primary Angiitis of the Central Nervous System (PACNS)	White and grey matter, leptomeningeal arteries	• infarcts, • intracranial hemorrhages • leptomeningeal gadolinium enhancement	• headache • cognitive impairment • seizures	[36]
Demyelinating disease	Multiple Sclerosis (MS)	Cortex, periventricular space, infratentorial space, spinal cord, optic nerve	• cortical atrophy, hyperintense lesions • hypointense lesions: “black holes” in predisposed areas	• muscle weakness • ataxia • diplopia, amaurosis fugax • cognitive dysfunctions • Lhermitte’s sign	[37]
	Acute Disseminated Encephalomyelitis (ADEM)	white and grey matter, spinal cord	• large, bilateral, asymmetrical lesions	• headache • pyramidal signs • ataxia • brainstem symptoms	[38]

### Cochlear microangiopathy

Inner ear vasculopathy is often associated with CNS or optic symptoms. However, this is not a common single symptom at the onset of the disease [9]. Since the cochlea is highly susceptible to ischemic damage, appropriate treatment should be started urgently to prevent irreversible hearing loss. Oral steroids are usually used; however, intratympanic administration also reduces the risk of deafness [8]. According to guidelines, treatment of inner ear vasculopathy requires intravenous methylprednisolone pulses for three consecutive days (1000 mg/day), oral prednisone, IVIG infusions, and preferably mycophenolate mofetil. Cyclophosphamide or tacrolimus is added in cases of subsequent exacerbations that occur despite prior treatment [9].

The woman no. 2 reported ENT symptoms associated with neurological disorders; however, ENT symptoms dominated during exacerbations (partial hearing loss, mild andchronic vertigo, and headache). She was treated with prednisone 60 mg orally, IVIG, and mycophenolate mofetil. The initial dose of mycophenolate mofetil is 500 mg twice daily, with a recommendation to increase the dose to 2 g after two weeks. However, the patient had adverse effects (nausea, abdominal pain) and reduced the dose to 500 mg (maximum tolerated dose). Fortunately, the disease stabilized with low-dose methylprednisolone and mycophenolate mofetil, so no further attempts were made to modify the medications or their doses.

In addition to immunosuppressive drugs, vitamin D was supplemented in each patient’s treatment. Adequate vitamin D levels have a beneficial effect on the immune system and prevent the development of osteoporosis. The role of vitamin D as an immune modulator is well documented, and its low levels correlate with the severity of autoimmune diseases [39–41]. Therefore, preventing vitamin D deficiency in our patients was an essential element of combined therapy.

Laboratory tests showed elevated inflammatory markers during exacerbations. We mainly observed elevated C-reactive protein (CRP) levels with a significantly increased concentration in patient 4. Interestingly, anemia during an exacerbation occurred only in this patient. Anemia of chronic diseases occurs in many rheumatic patients; however, it is not commonly observed in the course of SuS [42].

Serological analysis revealed low titers of antinuclear antibodies (ANA) in patients 1, 2, and 3. Pm-Scl antibodies were slightly positive in all our patients during the last hospitalization. Data on the type and level of antibodies in SuS differ between studies. In this syndrome, ANA may be slightly elevated in about half of the patients [43]. Some authors indicate the presence of antiphospholipid antibodies [10]. Jarius et al. described the presence of anti-endothelial cell antibodies (AECA), ANA, and ANCA antibodies. The authors noted that AECA antibodies seemed to be the most specific and could be detected in 30% of patients with definite SuS [44]. The clinical differences between our patients, as well as those described in the literature, prove that SuS is a heterogeneous disease without a pathognomonic immunological marker.

## Conclusions

The analyzed cases differ in clinical symptoms, confirming the heterogeneity of Susac syndrome. The triad of clinical symptoms (visual disorders, hearing loss, and neurological symptoms) is very characteristic of SuS; however, it was present in only one patient out of 4 analyzed cases (in the literature, it is described in 13–30% of patients).

Initial therapy with glucocorticosteroids and immunosuppressive drugs such as methotrexate, mycophenolate mofetil, sulfasalazine, and azathioprine causes disease remission, which proves their effectiveness and good tolerance. High doses of steroids shorten the time to achieve remission; unfortunately, their reduction in some patients was associated with disease exacerbation, which emphasizes the relapsing course of SuS. Moreover, in some patients, reducing the glucocorticosteroids dose below a certain level causes disease relapse (steroid dependence).

Although the initial treatment of SuS seems similar in each clinical manifestation, subtle differences should be emphasized. In the case of CNS symptoms, immunosuppressive drugs are always recommended as the primary treatment. In the current recommendations/suggestions, mycophenolate mofetil is mentioned as the immunomodulator of choice regardless of the type of clinical symptoms; however, other drugs also seem to be effective (methotrexate, azathioprine), both as monotherapy and in combination.

We hope that the described case series can increase the number of described patients, showing the heterogeneity of reported symptoms and the effectiveness of immunosuppressive therapy. The presented review of the case series emphasizes the importance of an individual therapeutic approach, which reflects the rules of personalized medicine.
